# Virtual Patient Technology: Engaging Primary Care in Quality Improvement Innovations

**DOI:** 10.2196/mededu.7042

**Published:** 2017-02-15

**Authors:** Amanda C Blok, Christine N May, Rajani S Sadasivam, Thomas K Houston

**Affiliations:** ^1^ Quantitative Health Sciences University of Massachusetts Medical School Worcester, MA United States; ^2^ Graduate School of Nursing University of Massachusetts Medical School Worcester, MA United States; ^3^ Preventative and Behavioral Medicine University of Massachusetts Medical School Worcester, MA United States; ^4^ Center for Healthcare Organization and Implementation Research Bedford Veterans Affairs Medical Center Bedford, MA United States

**Keywords:** virtual patients, interdisciplinary health teams, clinical staff engagement, environment design, health promotion, tobacco use cessation

## Abstract

**Background:**

Engaging health care staff in new quality improvement programs is challenging.

**Objective:**

We developed 2 virtual patient (VP) avatars in the context of a clinic-level quality improvement program. We sought to determine differences in preferences for VPs and the perceived influence of interacting with the VP on clinical staff engagement with the quality improvement program.

**Methods:**

Using a participatory design approach, we developed an older male smoker VP and a younger female smoker VP. The older male smoker was described as a patient with cardiovascular disease and was ethnically ambiguous. The female patient was younger and was worried about the impact of smoking on her pregnancy. Clinical staff were allowed to choose the VP they preferred, and the more they engaged with the VP, the more likely the VP was to quit smoking and become healthier. We deployed the VP within the context of a quality improvement program designed to encourage clinical staff to refer their patients who smoke to a patient-centered Web-assisted tobacco intervention. To evaluate the VPs, we used quantitative analyses using multivariate models of provider and practice characteristics and VP characteristic preference and analyses of a brief survey of positive deviants (clinical staff in practices with high rates of encouraging patients to use the quit smoking innovation).

**Results:**

A total of 146 clinical staff from 76 primary care practices interacted with the VPs. Clinic staff included medical providers (35/146, 24.0%), nurse professionals (19/146, 13.0%), primary care technicians (5/146, 3.4%), managerial staff (67/146, 45.9%), and receptionists (20/146, 13.7%). Medical staff were mostly male, and other roles were mostly female. Medical providers (OR 0.031; CI 0.003-0.281; *P*=.002) and younger staff (OR 0.411; CI 0.177-0.952; *P*=.038) were less likely to choose the younger, female VP when controlling for all other characteristics. VP preference did not influence online patient referrals by staff. In high-performing practices that referred 20 or more smokers to the ePortal (13/76), the majority of clinic staff were motivated by or liked the virtual patient (20/26, 77%).

**Conclusions:**

Medical providers are more likely motivated by VPs that are similar to their patient population, while nurses and other staff may prefer avatars that are more similar to them.

## Introduction

Engaging clinical staff in quality improvement interventions that promote clinical staff-patient discussions and referrals to health behavior change resources is key for health promotion, disease prevention, and disease management [1]. However, engaging staff is challenging. How do we activate clinical teams to adopt interventions that prescribe or introduce health promotion or health behavior activities to patients?

Interdisciplinary medical teams in the health care setting work collaboratively to provide comprehensive health services [2]. These teams commonly include medical providers, nurse professionals, patient care technicians, social workers [3], and increasingly include administrative staff for enhanced communication within and between clinical teams [4]. Increasingly, interventions are targeted to motivate clinical teams to engage patients in health-promoting behaviors [5]. Techniques to motivate physicians, nurses, and primary care staff to encourage patient health-promoting behaviors traditionally include reminders and performance feedback [6,7]. While these techniques are successful in the short term, they do not provide continuous reminders to cue behavior or sustainably engage providers for the long term. Clinical staff often do not see the outcomes of their health promotion activities on patients, potentially leading to a lack of positive feedback and reinforcement and lack of sustainability of quality improvement initiatives. Novel methods of engaging clinical staff in informatics innovations that support quality improvement could enhance the feeling that clinical staff are making an impact and improving the health of their patients.

Relational agents or avatars, digital and animated representations of people, are a newer form of engagement and motivation. Virtual patient (VP) avatars have been used to motivate healthy behaviors in patients, typically as patient coaches [8-11]. For example, a depression self-management intervention for young adults using virtual health care providers and virtual coaches significantly decreased depression symptoms [12]. The medical and nursing disciplines have used VPs to improve education on critical thinking [13,14]. To date, VPs have not been used in the practice setting to change provider behavior and encourage quality improvement initiatives. In this context, the avatar is present on staff computer screens as a continual cue to perform a behavior, such as counseling a patient to quit tobacco. The avatar intrinsically motivates staff to introduce patients to healthy behaviors, with the avatar’s facial expression and narrative improving with greater amounts of positive staff behavior. However, these avatars have not been rigorously evaluated in the context of changing clinical practice patterns on the provider side. To evaluate the feasibility and potential for VPs in the clinical context, we developed and deployed 2 VPs within the context of a practice-level quality improvement program for smoking cessation.

This report describes the use of and reaction to the virtual patients (Bob and Susie) among the clinical staff of 87 primary care practices. In primary care, people in different staff roles usually have different technology preferences [15]. Thus, we were interested in the influence of staff role type on preferences for engaging with the VPs, as well as the influence of VP preference on clinical staff performing the activities in the smoking cessation quality improvement programs during a 3-month follow-up period. Our research objectives were to (1) determine VP preference by clinic staff role and primary care practice characteristics, (2) determine the influence of these characteristics and VP preference on clinical staff engagement with the quality improvement program (as described below, clinical staff were encouraged to refer patients to an online Web-assisted tobacco intervention as a part of the quality improvement program), and (3) explore perceived usefulness and motivation VP preference had on engagement and examine differences by staff role among practices with high levels of engagement in the quality improvement program (high patient referral rates). Examining the differences in technology use and preferences among primary care staff will enable further development of VP improvements that motivate staff to adopt and sustain quality improvement programs.

## Methods

### Study Description

The VP study was a prospective, observational study of physicians, nurses, and other primary care staff and their engagement with a longitudinal quality improvement study that used VPs to enhance engagement. The VPs were deployed in the context of a larger practice improvement program, the “Quality Improvement in Tobacco-Provider Referrals and Internet-Delivered Microsystem Optimization (QUIT-PRIMO)” trial [16]. The goal of QUIT-PRIMO was quality improvement in tobacco control, using a program assisted by a clinic-level ePortal to engage and remind the clinical staff of health care practices to recommend and refer their tobacco-smoking patients to a patient-level Web-assisted tobacco intervention. The results of the QUIT-PRIMO ePortal trial were previously reported [17]. The study was approved by the institutional review board, and the protocol was overseen by a data safety and monitoring board.

### Clinic-Level ePortal Quality Improvement Program Overview

A total of 76 practices received the technology-assisted quality improvement program. The quality improvement program used a Web-based system (ePortal) to have practices enter their patient email addresses (with patient consent) and electronically refer patients to the patient-level Web-assisted tobacco intervention. After their visit, patients received up to 10 automated email reminders (personalized by the medical provider) to remind the smokers to participate in the Web-assisted tobacco intervention the clinic had recommended. The clinic-level ePortal quality improvement program resulted in nearly threefold greater patient participation (31%) than the rate in comparison practices using paper brochures to refer patients (11% patient participation in the Web-assisted tobacco intervention). Over 2000 patients were referred using the ePortal.

The ePortal home page included a VP. Each VP was created to assist the implementation of the quality improvement program (staff referrals of patients who are current smokers to the Web-assisted tobacco intervention). As described below, clinical staff were allowed to select their preferred VP. Data on participant selection of a VP character and referral rates were collected through the online database. Participants from clinics with a high referral rate (20 referrals or more) were targeted for an interview as a study of positive deviants, with questions pertaining to components of the QUIT-PRIMO trial including attitudes toward VPs.

### Virtual Patient: Participatory Design Approach

**Figure 1:**
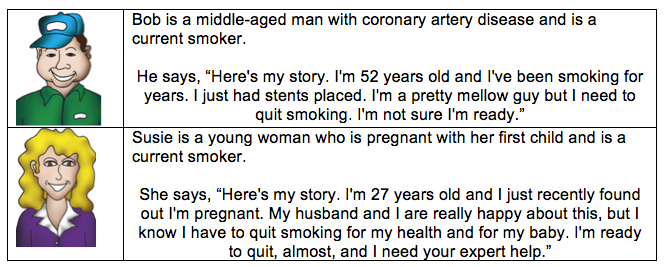
Virtual patient character description.

We used a systematic participatory design process to create the VPs. A professional artist initially developed 6 VP characters that were pilot-tested with a group of health care providers and other clinical staff (N=8) at an academic primary care practice. The VPs were designed to motivate users by transforming their facial expression and narrative as more smokers were referred using the ePortal (Multimedia Appendix A). Based on feedback from clinical staff, 2 avatars were selected. These 2 avatars received positive qualitative comments from providers and staff, and no staff felt that these VPs were disliked. Although some other VPs were liked by some providers, they also received negative comments (like “not realistic” or “overly healthy” or “confusing” story). Providers stated that they selected the 2 characters because they most represented their patients and felt the artistic rendering was a good fit for patients’ stories. Providers also claimed to feel an empathetic connection to these 2 characters. See Figure 1 for a description of the two VPs (Bob and Susie) used in the ePortal quality improvement program.

The VPs were designed to change their story as the clinical staff used the ePortal. The more referrals of actual patients (meaning clinical staff entered the patient email address into the ePortal system so that the patient would receive follow-up reminders), the more the VP progressed through their own quitting tobacco story. There were 16 transformations of facial expression and/or verbal feedback in text form used for Bob and Susie. Multimedia Appendix 1 gives 5 examples each of Bob and Susie’s story as they progress through quitting related to the amount of referrals clinical staff enter online for their patients.

### Recruitment

Each primary care practice participating in the quality improvement program was asked to identify 2 clinical staff to serve as implementation coordinators. These coordinators could include physicians, nurses, primary care technicians, secretaries, receptionists, and managers. These staff logged on to the ePortal quality improvement program where they received education about advising current smokers to quit and an online form that they could use to e-refer patients. They selected one of the VPs (Bob or Susie) to use for the course of the study.

### Measures

Practices were recruited using mass mailing from a mailing list of practices until the sample size (76 practices) was achieved. During recruitment, practices completed a survey assessing practice-level characteristics, including region of the country. Clinical staff registering on the ePortal quality improvement program consented online and then completed an online survey that assessed clinic staff type, demographic information, and technology use. VP preference (Bob or Susie) was gathered when clinical staff registered in the online database (referasmoker.org). Documentation use of the ePortal by clinical staff was collected throughout the study on the online database. We interviewed staff of the practices who referred 20 or more smokers to the ePortal.

Several variables were constructed. Age was dichotomized on the 50th percentile for descriptive purposes. Categorical variables were recoded into dummy variables to better understand if any individual staff or practice characteristics affected VP preference. Dummy variables were created for staff role, practice type, practice region, and participant race for bivariate analyses with VP character choice. A total Web technology use score was calculated as the sum of 6 markers of Internet use collected at baseline (dichotomous variables for each function: using the Internet, searching for information, reading information, using email, using online social media, and input-based use). From this total score, a dichotomous variable of low or moderate technology use was constructed, with low use indicating 2 kinds of Web technology use or fewer and moderate use as 3 or more kinds. A categorical variable of referrals was created, including 3 categories: (1) no referrals, (2) referrals under the set goal of 20 referrals, and (3) referrals meeting or exceeding the goal of 20 or more.

In addition to the quantitative data above, we conducted a follow-up qualitative study of positive deviants. Positive deviants are people who have more positive outcomes than others within the same context and resources available [18]. In this study, we defined positive deviants as clinical staff who had used the ePortal over 20 times to refer patients. Participating clinical staff within primary care practices that had overall referral rates higher than 20 were selected for interviews with both closed- and open-ended questions. A multiple choice question “How did you feel about the virtual patient?” with options of “It made me want to come back to website to refer patients,” “I liked it,” “I found it annoying,” “I wanted to skip over it,” and “other” was used to assess staff perceptions of VP technology usefulness. Subsequently, there was an opportunity to comment further in an open-ended way, and those who selected “other” were elicited for more information.

### Statistical Analysis

Descriptive statistics were used for sample description. The impact of clinic staff roles on VP preference (Bob or Susie? Objective 1), VP influence on clinical staff use of the ePortal quality improvement program (Objective 2), and motivation to use the VP (Objective 3) were analyzed using the chi-square test due to their categorical nonparametric nature. For multivariable analyses, a survey analysis strategy (Stata svyset, StataCorp LLC) was used to account for the survey design sampling method of multiple clinical staff at each primary care site. Categorical variables were included in models as indicator variables. A logistic regression was performed to determine the influence of practice type characteristics (internal medicine or family practice, region of the country), professional characteristics (clinic staff role), and personal characteristics (age, gender, race) on VP selection (Objective 1). A logistic regression was performed to determine whether the number of e-referrals was influenced by VP selection or personal or practice type characteristics (Objective 2). Qualitative results were coded and summarized (Objective 3). Stata 12.1 (StataCorp LCC) software was used for all analyses, with *P* values of less than .05 considered significant.

## Results

### Characteristics of Primary Care Staff and Their Use of Technology

Table 1 provides characteristics for 146 primary care staff from 76 practices. Clinic staff included medical providers (35/146, 24.0%), nurse professionals (19/146, 13.0%), primary care technicians (5/146, 3.4%), receptionists (20/146, 13.7%), and managerial staff (67/146, 45.9%). The majority of the sample was female (121/146; 82.9%), with almost two-thirds of the medical providers being male (22/35; 62.8%) and almost all nonmedical providers female (108/111; 97.3%). Web technology use by primary care staff varied by staff role, with medical providers having the highest use of technology (mean 5.0, SD 1.2) and patient care technicians having the lowest (mean 3.2, SD 2.7) (Table 1).

**Table 1:** Clinic and staff characteristics.

Characteristics		Total (N=146) n (%)
**Staff role**		
	Medical providers^a^	35 (24.0)
	Nurse professionals^b^	19 (13.0)
	Patient care technician	5 (3.4)
	Receptionist/secretary	20 (13.7)
	Managerial staff	67 (45.9)
**Practice type**		
	Internal medicine	63 (43.2)
	Family medicine	81 (55.5)
	General practice	2 (1.4)
**Practice region**		
	Northeast	45 (30.8)
	Midwest	27 (18.5)
	West	32 (21.9)
	Southeast	42 (28.8)
**Participant age**		
	<35 years	59 (40.4)
	≥35 years	87 (59.6)
**Participant gender**		
	Male	25 (17.1)
	Female	121 (82.9)
**Participant race**		
	White	100 (68.5)
	Black	20 (13.7)
	Other race	26 (17.8)

^a^Medical providers include medical doctors, doctors of osteopathic medicine, and physician assistants.

^b^Nurse professionals include registered nurses, licensed practical nurses, and nurse practitioners.

### Factors Associated with Virtual Patient Choice (Objective 1)

In these 76 primary care practices, 61% (89/146) of clinic staff chose Bob and 39% (57/146) chose Susie. All male clinic staff (providers, nurses, and other staff) selected the male VP, Bob, as their VP for the study (25/146). In bivariate analyses, medical provider role (*P*<.001) and clinical staff age greater than 35 (*P*<.001) were more likely to select the older, male VP than other participants (Table 2). These associations persisted in multivariable regression (Table 3). In multivariate analysis, medical providers were 96.9% less likely to choose Susie (odds ratio [OR] 0.031; CI 0.003-0.281; *P*=.002) than secretarial or managerial staff. In the same model, clinical staff older than 35 years were 58.9% less likely to select the young, female VP (OR 0.411; CI 0.177-0.952; *P*=.038), even when controlling for all other characteristics.

**Table 2:** Bivariate associations of virtual patient preference by clinical and demographic characteristics.

		Bob N=89 (61.0%) n (%)	Susie N=57 (39.0%) n (%)	*P* value^a^
**Staff role**				
	Medical providers	34 (97.1)	1 (2.9)	.000
	Nurse professionals	13 (68.4)	6 (31.6)	.475
	Patient care technician	4 (80.0)	1 (20.0)	.374
	Receptionist/secretary	8 (40.0)	12 (60.0)	.039
	Managerial and other staff	30 (44.8)	37 (55.2)	.000
**Practice type**				
	Internal medicine	49 (60.5)	32 (39.5)	.838
	Family medicine	39 (61.9)	24 (38.1)	.898
	General practice	1 (50.0)	1 (50.0)	.749
**Practice region**				
	Northeast	27 (60.0)	18 (40.0)	.874
	Midwest	17 (63.0)	10 (37.0)	.813
	West	19 (59.4)	13 (40.6)	.835
	Southeast	26 (61.9)	16 (38.1)	.882
**Participant age**				
	Age <35	24 (40.7)	35 (59.3)	.000
	Age ≥35	65 (74.1)	22 (25.3)	
**Participant gender**				
	Male	25 (100.0)	0 (0.0)	.000
	Female	64 (52.9)	57 (47.1)	
**Participant race**				
	White	69 (61.1)	44 (38.9)	.962
	Black	9 (45.0)	11 (55.0)	.115
	Other race	11 (84.6)	2 (15.4)	.067
**Technology use**				
	Low technology use	33 (54.1)	28 (45.9)	.150
	High technology use	56 (65.9)	29 (34.1)	

^a^*P* values express differences between categories using dummy variables.

**Table 3:** Virtual patient preference by clinical and demographic characteristics using multivariate analysis.

Characteristics		Model		
Variable (reference group)		Odds ratio	95% CI	*P* value
**Staff role (managerial staff)**				
	Medical providers	0.031	0.003-0.281	.002
	Nurse professionals	0.413	0.104-1.634	.205
	Patient care technician	0.219	0.006-7.411	.394
	Secretarial staff	1.164	0.349-3.879	.802
**Practice type (family medicine)**				
	Internal medicine	0.788	0.331-1.875	.585
	General practice	1.155	0.395-3.375	.790
**Practice region (Northeast)**				
	Midwest	0.998	0.289-3.441	.997
	West	0.786	0.264-2.340	.663
	Southeast	0.410	0.140-1.198	.102
**Participant age (<35 years)**				
	≥35 years	0.411	0.177-0.952	.038
**Participant race (white)**				
	Black	2.427	0.582-10.10	.220
	Other race	0.332	0.062-1.771	.194
**Technology use**				
	High technology use	0.937	0.403-2.181	.879
Constant		2.860	0.914-8.952	.071

### Influence of Virtual Patient and Staff Characteristics on eReferrals (Objective 2)

Staff role, practice type, and race were significant in predicting referrals (Table 4, Model 2). Importantly, the VP character type was not significant in influencing e-referrals to an online tobacco cessation intervention (Table 4, Model 1), even when controlling for other staff and practice characteristics (Table 4, Model 2). Medical providers were 4 times more likely than clinic managers to refer smokers to the online tobacco cessation intervention (OR 4.319; CI 1.261-14.797; *P*=.020). Staff who work in internal medicine were more than twice as likely as those working in a family medicine clinic to refer patients (OR 2.215; CI 1.040-4.719; *P*=.040). Staff who were black were 72.3% less likely to refer patients than staff who were white (OR 0.279; CI 0.091-0.854; *P*=.026).

**Table 4:** Referrals to Web-assisted tobacco intervention by clinical and staff characteristics using multivariate analyses.

		Model 1			Model 2		
Variable (reference group)		OR	95% CI	P value	OR	95% CI	P value
**Virtual patient** (Bob)							
	Susie	1.072	0.562-2.045	.830	2.000	0.819-4.868	.127
**Staff role (managerial staff)**							
	Medical providers				4.319	1.261-14.797	.020
	Nurse professionals				1.417	0.433-4.641	.561
	Patient care technician				0.130	0.008-2.180	.154
	Secretarial staff				0.566	0.187-1.714	.310
**Practice type (family medicine)**							
	Internal medicine				2.215	1.040-4.719	.040
	General practice				1.000		
**Practice region (Northeast)**							
	Midwest				0.795	0.290-2.177	.652
	West				3.176	0.997-10.115	.051
	Southeast				2.241	0.818-6.138	.115
**Participant age (<35 years)**							
	≥35 years				0.790	0.314-1.990	.614
**Participant race (white)**							
	Black				0.279	0.091-0.854	.026
	Other race				1.191	0.345-4.111	.779
**Technology use**							
	High technology use				0.857	0.392-1.872	.696
Constant		1.282	0.832-1.975	.256	0.569	0.148-2.190	.408

### Staff Perceptions of Virtual Patient Technology Usefulness (Objective 3)

In high-performing practices that referred 20 or more smokers to the ePortal (13/76), the majority of clinic staff reported they were motivated by or liked the VP (20/26, 77%). Two-thirds of secretarial staff were motivated by the VP to refer patients (4/6, 67%). While medical providers were less likely to agree they were motivated by the VP (2/7, 29%), most medical providers liked the VP (4/7, 57%) (Table 5). One medical provider found the VP annoying, but no clinical staff reported they wanted to skip over the VP. A total of 5 clinical staff selected “other” in response to the categorical VP impression question (5/26, 19%). These staff commented they had low personal e-referral experience (2/5), did not use the e-referral system (2/5), or “didn’t notice” the VP (1/5).

**Table 5:** Motivation from and acceptability of the virtual patient by clinical staff role.

		VP^a^ motivated e-referrals n (%)	Liked VP n (%)	VP did not motivate or did not like VP n (%)	Total n
**Staff role**					
	Medical providers	2 (29)	4 (57)	1 (14)	7
	Nurse professionals	1 (50)	1 (50)	0 (0)	2
	Patient care technician	0 (0)	0 (0)	1 (100)	1
	Secretarial staff	4 (67)	1 (17)	1 (17)	6
	Managerial and other staff	4 (40)	3 (30)	3 (30)	10
**Total**		11 (42)	9 (35)	6 (23)	26

^a^VP: virtual patient.

## Discussion

In 76 clinical practices, we found strong differences in preference for VP by clinical staff role. Clinical staff in different roles have different technology preferences in technology innovations. For example, in a study of 9 information technology innovations for hospice use, researchers found that patients, physicians, nurses, managers, and others each preferred different innovation structures [15]. Thus, we were interested in the influence of staff role type on preferences for engaging with VPs and also in the influence of VPs on staff referrals to the Web-assisted tobacco intervention. We found distinct VP preferences mediated by clinical staff characteristics and positive impressions of VPs as agents to engage staff in quality improvement, although preference for individual VP did not influence participation in the quality improvement program. Below, we place these principal results into context.

### Principal Findings

#### Staff Virtual Patient Preference (Objective 1)

In this study, the choice of VP varied based on staff role. Medical providers chose the VP that most fit their patient population, while administrative staff preferred the same-gender VP. This finding may indicate that medical providers are more likely motivated by VPs like their patient population, while other staff are more motivated by VPs that are similar to them. Health care providers have been shown to select VPs in virtual telemedicine to represent what characteristics patients are more likely to prefer or respond to, such as gender and race [19]. This phenomenon of selecting these characteristics to elicit a positive response by patients likely extends into motivating themselves to engage patients in health behavior change. Our findings extend this research into the realm of quality improvement.

#### Referrals to Web-Assisted Tobacco Intervention by Clinical and Staff Characteristics (Objective 2)

The VP avatars did not differ in influence of staff to refer patients to the Web-assisted tobacco intervention, controlling for all other clinic and staff characteristics. This is an important finding, as VP preference did not influence staff decisions to refer patients over other inherent characteristics of who they are and the clinical setting where they work. VPs’ influence on provider performance needs further study. Developing and tailoring VPs to provider characteristics is in its infancy for motivational behavior.

Personal and practice characteristics were significant in predicting referrals to the Web-assisted tobacco intervention. Medical providers were the most likely to refer patients to an online intervention compared to other staff. Focusing on these staff members to increase referral rates needs to be examined. In addition, determining strategies in conjunction with VPs to encourage staff on the clinic team to use referrals should be explored. Different users have different needs during the implementation of innovations [20]. Further research to determine what VP characteristics appeal most to health care staff will assist in using this motivational technology to make an impact on health promotion efforts.

#### Perceptions of Virtual Patient Technology Usefulness (Objective 3)

Medical providers, nurses, and secretarial staff were more likely than technicians and managerial staff to find the VP useful and motivating for e-referrals. The differences in priorities among staff roles point to different technology preferences and needs during the implementation of innovations [20]. Familiarity with technology is known to influence clinical staff attitudes toward new technology use [21,22]. Technology use is higher among medical providers than administrative staff, which may indicate unfamiliarity and low use of the intervention. Our study of positive deviants also indicates those who were not motivated by the VP or did not like the VP were unfamiliar with the e-referral system and had low personal use of this technology. Therefore, a more thorough introduction to the VP and further training for clinical staff may create more positive perceptions of VP usefulness. To assist all staff members in participating in technology use, Das and colleagues recommend development of education and guidelines targeted to this group. Such guidelines can outline how to best communicate with and facilitate staff online use. This is a stepping stone toward building organizational infrastructure and incentives which can then facilitate Web technology use [23]. The differences in preference by clinical staff role point to the need for further research to determine characteristics that motivate each role for enhanced health promotion efforts.

### Limitations

There were limitations to this study. This sample of primary care staff was primarily female. A majority of managerial, secretarial, and nursing staff were women. Overall, there was a low number of male participants, and no male nurses or secretaries were included, limiting the exploration of factors that influence male staff VP selection. However, these staff roles are known to have limited numbers of men. Both VPs in the medical setting were white, limiting the analysis of the influence of demographics on VP selection (aim 2). Finally, the subanalysis of staff perceptions of VP technology usefulness in high-referring practices included a relatively low number of participants, limiting the strength of findings on health care provider perceptions of usefulness (aim 3).

### Comparison With Prior Work

Clinical staff often do not see the impact of their health promotion activities on their patients, which leads to a lack of positive feedback or reinforcement for these activities [16,24]. This phenomenon may contribute to a lower sustainability of quality improvement initiatives in the clinical setting. Novel methods of engaging clinical staff in activities that support health promotion have an opportunity to enhance provider feelings of impact on patient health. Similarly, performance feedback for initiatives has been reported to increase clinical staff pride in their personal or their practice’s achievement [25]. The VP transformation to better health coordinated with the provider behavior transformation to increased smoker referrals is a visual form of performance feedback that taps into providers’ intrinsic motivation of effective patient care.

VPs are a novel informatics innovation to intrinsically motivate clinical staff to change their behavior. A prevalent problem in the clinical setting is difficulty motivating clinical staff to incorporate a new task into their clinical workflow [26]. Solutions for motivation in health care have focused primarily on extrinsic sources, such as financial incentives, to change practice behaviors. These extrinsic incentives have crowded out intrinsic motivators, such as patient improvement. However, extrinsic incentives do not promote sustainability in practice change, as they commonly expire. Intrinsic motivation, the satisfaction of doing a job well with good outcomes as the reward, is just starting to be harnessed for provider change [27].

Motivational interviewing (MI) has been proposed to motivate health care providers to adopt evidence-based practices. As a tool to assist people in resolving ambivalence about change by incorporating principles that parallel Roger’s diffusion of innovation theory [28], its purpose aligns with provider motivation to change a practice. MI has been used in webinars to implement an intervention [28]. Similarly, VPs could continue to be developed to incorporate principles of MI to enhance its effect on provider behavior. Verbal or written messages from VPs could incorporate elements of MI that could be used as a component for an effective strategy to change provider behavior.

A barrier to VP effectiveness or usefulness has been a lack of realism in both the context of clinical staff education and patient intervention [11,29,30]. Unrealistic visual components for patient assessment was a detracting factor in education and was a focused part of the study. Realistic features pertinent to the focus of the intervention would enhance the perceived usefulness of the VP. For example, if the focus of the VP intervention is on clinical staff or patient behavior, then the messages and responses of the VPs related to the behavior targeted for change need to be realistic. Our VPs are cartoon representations of patients that providers chose as realistic representations of patients in their practice setting. While we did not ask about perceived realism of the VPs to participants, none of the clinical staff reported a lack of realism as a criticism. Further development and testing of characters and messages for VPs to change behavior is needed in the strategy for clinical staff behavior change.

### Conclusions

Clinical staff personal and professional characteristics influence VP character preferences and e-referral rates. Administrative staff selected the VP that was same-gender, while medical providers were more likely to select different-gender VPs. Clinical staff preferred VPs similar to their patients and administrative staff preferred staff similar to themselves, which may indicate the need for tailoring VPs according to staff role. VP character preference did not predict staff referrals to an online behavioral intervention in this study. However, high-referring primary care practice clinical staff reported they were motivated by VPs, indicating VPs as a potentially successful strategy for quality improvement programs in some practice settings. Therefore, further development of the VP characters and facilitative strategies need to be explored. Now that the feasibility of VPs in the context of quality improvement has been preliminarily tested, our future work will conduct randomized experiments to test the impact of the addition of VPs to traditional motivational components of quality improvement programs.

## Appendix

Multimedia Appendix 1 Virtual patient character transformation.

## Abbreviations

MI: motivational interviewing
OR: odds ratio
QUIT-PRIMO: Quality Improvement in Tobacco-Provider Referrals and Internet-Delivered Microsystem Optimization
VP: virtual patient
