# Supplementary material for: Virtual Patient Technology: Engaging Primary Care in Quality Improvement Innovations
Source: JMIR Med Educ. 2017 Feb 15;3(1):e3. doi: 10.2196/mededu.7042 (PMC5332834; doi:10.2196/mededu.7042)
Supplement: Multimedia Appendix 1 [file mededu_v3i1e3_app1.pptx]

## Slide 1
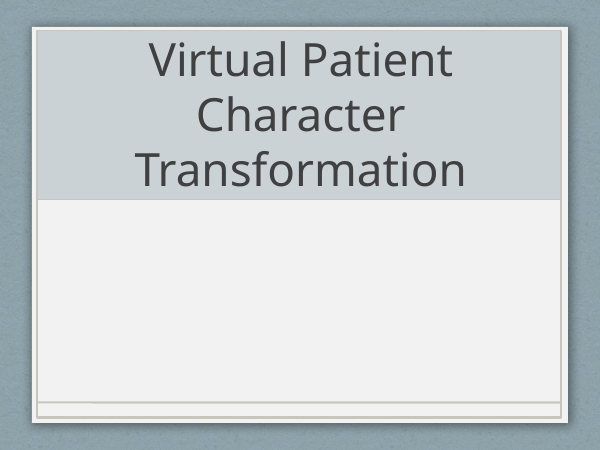

# Virtual Patient Character Transformation

## Slide 2
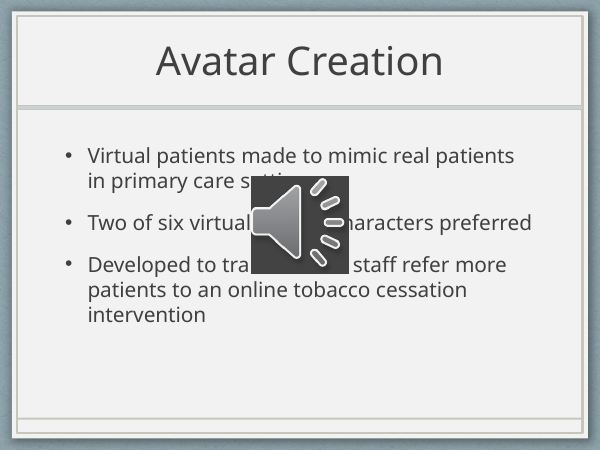

# Avatar Creation
Virtual patients made to mimic real patients in primary care setting
Two of six virtual patient characters preferred
Developed to transform as staff refer more patients to an online tobacco cessation intervention

## Slide 3
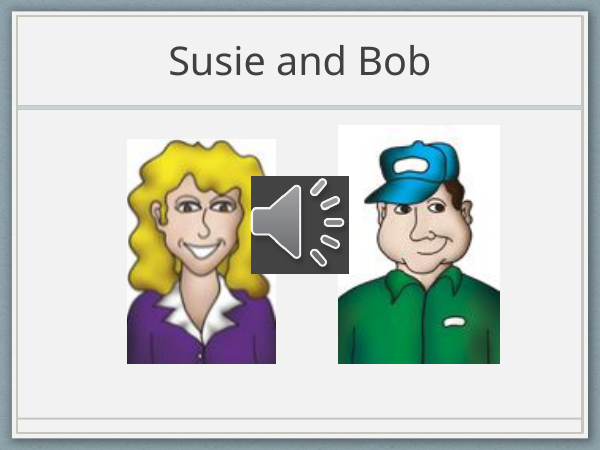

# Susie and Bob

## Slide 4
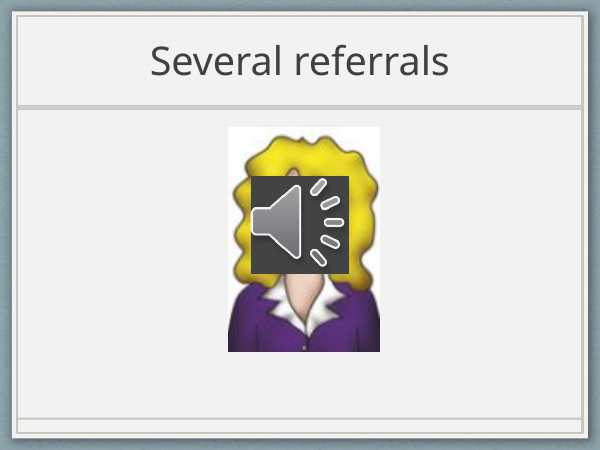

# Several referrals

## Slide 5
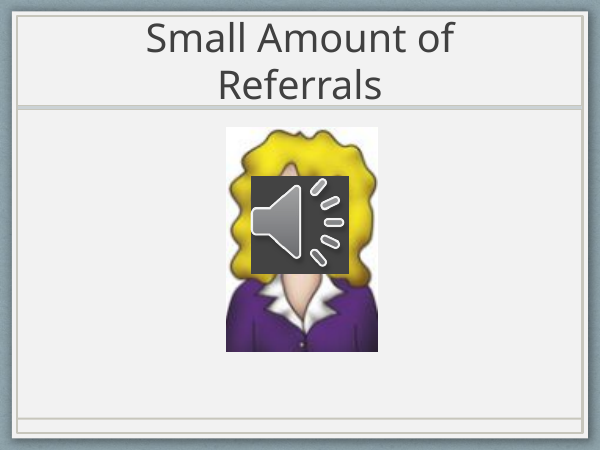

# Small Amount of Referrals

## Slide 6
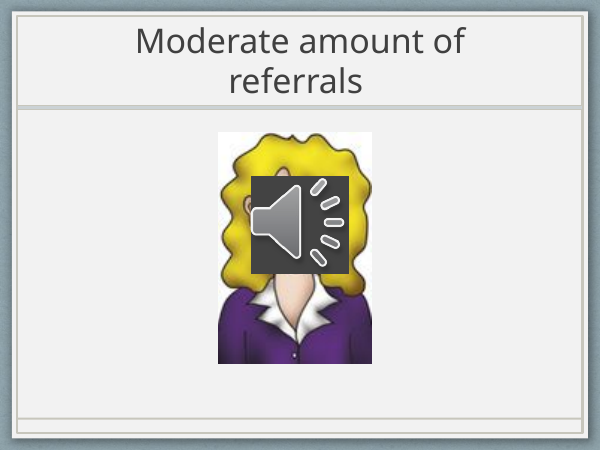

# Moderate amount of referrals

## Slide 7
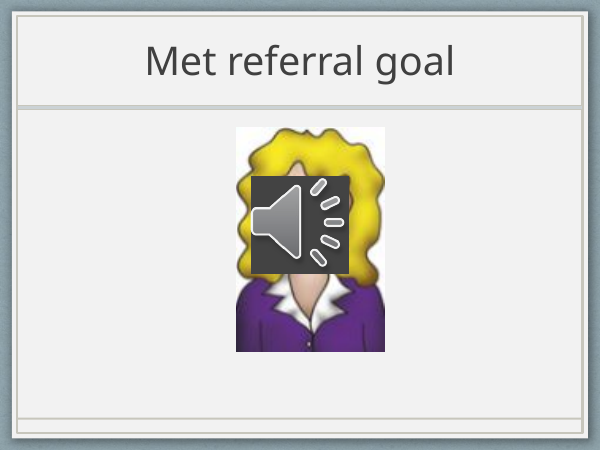

# Met referral goal

## Slide 8
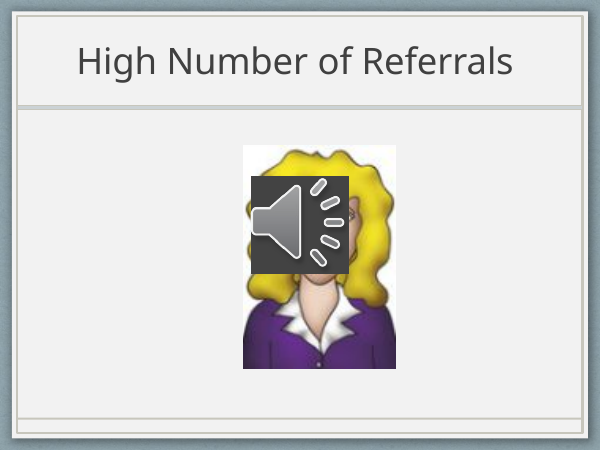

# High Number of Referrals

## Slide 9
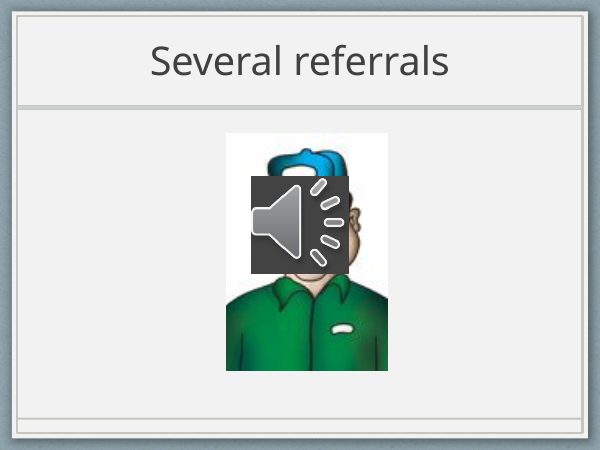

# Several referrals

## Slide 10
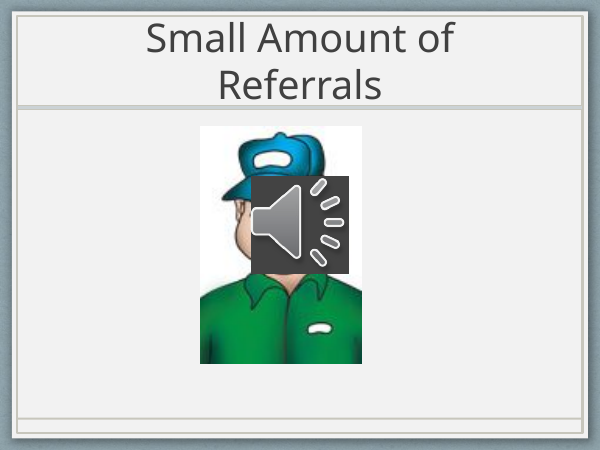

# Small Amount of Referrals

## Slide 11
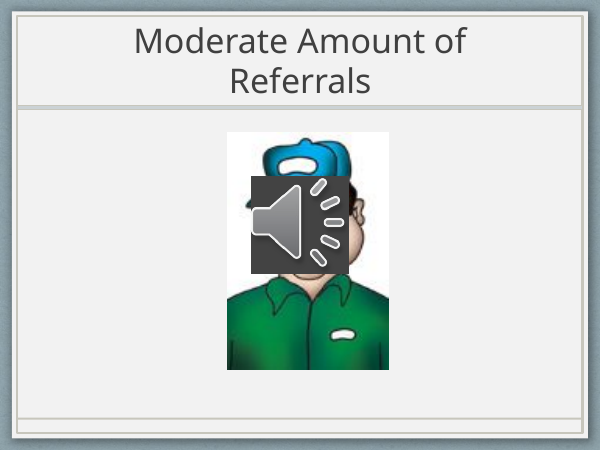

# Moderate Amount of Referrals

## Slide 12
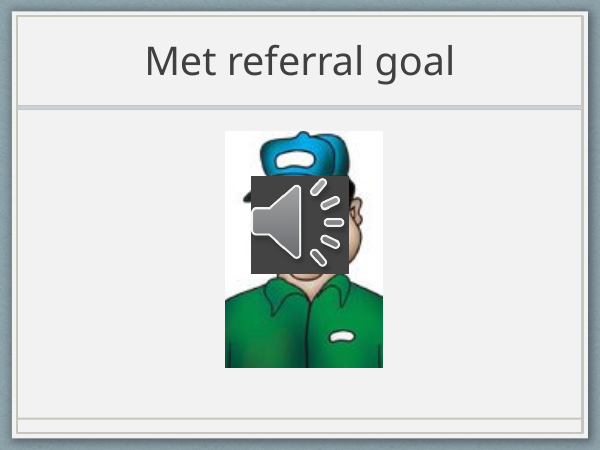

# Met referral goal

## Slide 13
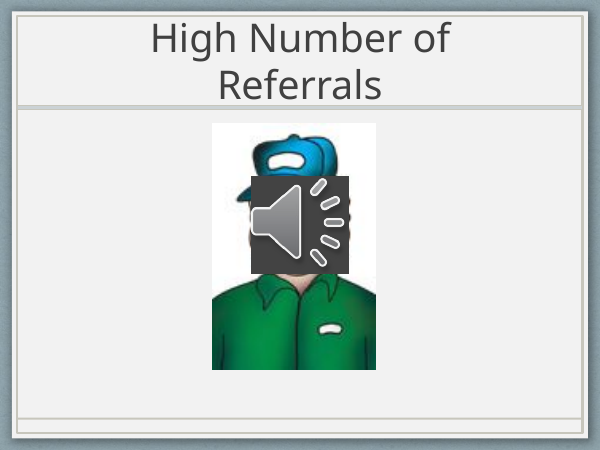

# High Number of Referrals

## Slide 14
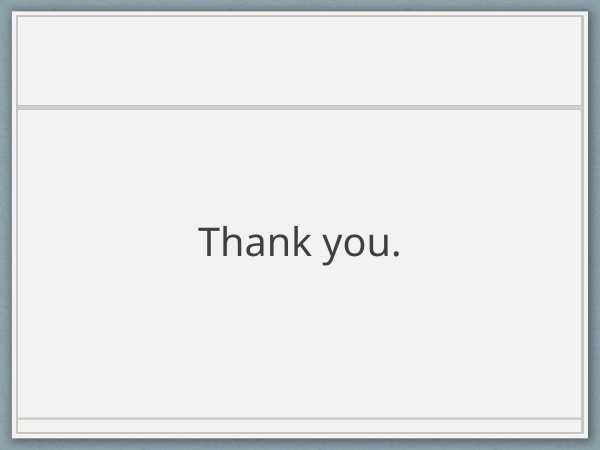

# Thank you.
